# The Implementation of Application Software to Improve Verbal Communication in Children with Autism Spectrum Disorder: A Review

**DOI:** 10.3390/children8111001

**Published:** 2021-11-03

**Authors:** Marlyn Maseri, Mazlina Mamat, Hoe Tung Yew, Ali Chekima

**Affiliations:** Electronic Engineering (Computer) Program, Faculty of Engineering, Universiti Malaysia Sabah, Kota Kinabalu 88400, Sabah, Malaysia; marlyn.maseri@gmail.com (M.M.); htyew@ums.edu.my (H.T.Y.); chekima@ums.edu.my (A.C.)

**Keywords:** autism spectrum disorder, apps, intervention, verbal communication

## Abstract

Autism-assistive apps offer therapists and caregivers new approaches for educating and assisting individuals with autism spectrum disorder (ASD), mainly in social interaction. Even though these apps are deemed effective, they are not. These autism-assistive apps are not highly customizable, which limits their usefulness. This article examined the application software that was applied to encourage verbal communication in the intervention for children with ASD. The aim was to determine the minimum requirements for a verbal communication intervention app that adequately satisfies children with ASD, caregivers, and therapists. Databases were searched, including Scopus, Springer, PubMed, Education Resources Information Centre, and Google Scholar, with the following free-text terms combining Boolean operators: autism, children, intervention, verbal communication, software, app, and technology. A total of fifteen studies were found relevant, and the following information was collected: participant characteristics, information on the devices and apps, target behaviors, intervention procedures, and intervention outcomes. The findings suggest that the autism-assistive apps effectively improve verbal communication of children with ASD. For that, the apps should be attractive and engaging to the children with ASD, able to identify the child’s capability and suggest appropriate lesson activities, as well as encompass specific learning outcomes with multilevel lesson strategy. The apps should also use systematic evidence-based intervention procedures in the activities, be able to evaluate the child’s learning progress, and allow caregivers or therapists to keep track of application usage and performance. The use of apps in intervention does provide many benefits. However, they should never replace qualified therapists. App-based interventions make home-based treatment more focused, systematic, and economical.

## 1. Introduction

Autism was classified as a developmental disorder by Leo Kanner in 1948. Since then, researchers have shown an interest in autism, resulting in continuous changes in the diagnostic criteria of autism [1]. In May 2013, the American Psychiatric Association released the fifth edition of the Diagnostic and Statistical Manual of Mental Disorders (DSM-5), which included predominant changes to the criteria that are used to diagnose autism. These new criteria have two impairment domains: (1) social interaction and social communication; and (2) restricted interests and repetitive behaviors. Additionally, Autistic Disorder, Asperger’s Disorder, and Pervasive Developmental Disorder—Not Otherwise Specified (PDD-NOS) are categorized into one umbrella term: “Autism Spectrum Disorder, ASD” [2].

The symptoms of autism spectrum disorder (ASD) usually present in the early developmental period. Individuals with ASD commonly struggle to communicate, such that they experience difficulty initiating conversations socially. They also have difficulty responding to communicative bids of others and engaging in reciprocal exchange [3]. Approximately 25 to 61% of children with ASD have little or no functional speech [4], while around 25 to 50% still have not developed language even by reaching 10 to 13 years old [5]. Learning basic communication skills is crucial as communication is an essential part of everyday life; communication is needed to exchange messages, thoughts, feelings, and information with other people. Generally, communication is comprised of speech, vocalizations such as sounds and shouts, body languages such as facial expressions and posture, sign language or the exchange of pictures, the use of communication devices, and writing [6].

In this modern era, individuals with ASD benefit from technology. Researchers are convinced that technology could help individuals with ASD improve their quality of life [7,8,9,10]. Several review papers have discussed the effectiveness of using technology-based intervention to improve communication skills for individuals with ASD. Digennaro, Hyman and Hirst focused on studies that used technology for social skills intervention and suggest that technology is helpful to teach social skills for children with ASD [11]. Ramdoss et al. reviewed the use of computer-based interventions (CBI) to teach communication skills to children with ASD [12]. They concluded that integration of technology in intervention is a promising practice for improving vocal and non-vocal communication. Wainer and Ingersoll claimed that an innovative multimedia program could be a good strategy for delivering the direct intervention to teach language, emotion recognition, or social skills to children and adults with ASD [13]. Kagohara et al. concluded that handheld devices, such as iPods, iPhones, and iPads can be used within educational programs that are targeting academic, communication, employment, and leisure skills for individuals with ASD [14]. A systematic review by Still et al. suggested that high-tech devices (e.g., smartphone technology) could be applied as augmentative and alternative communication (AAC) devices for individuals with ASD [15]. In their review, Kristy, Teresa, and David echo these findings by stating that the AAC interventions are helpful to assist children with ASD to communicate, particularly to request preferred items and activities [16]. Heath et al. proposed that technology could alleviate the treatment cost while allowing more significant support for individuals that are interested in the treatments [17]. In addition, the positive effect of the technology-based intervention on children with ASD is prolonged. Post-intervention analysis showed that the language and social communication skills of children with ASD were maintained for at least a year after ceasing the technology-based intervention [18]. 

The critical factor that makes technology successful is the application software (‘apps’) installed. Designers must ensure that the apps have high interactivity and be of good quality, meet users’ needs, and have an effective learning framework [19,20]. It is challenging to find one that is suitable for ASD intervention [21,22]. From the literature, it can be said that no comprehensive intervention apps for individuals with ASD are available. All related publications used the AAC, speech generating device (SGD), or educational apps as the tools to deliver the intervention. This is due to the fact that ASD is a spectrum, and the needs of individuals vary. An intervention app should be designed as completely as possible, covering lessons from the lowest to the highest difficulty levels while allowing caregivers or therapists to select the lesson plan or add new activities. Considering the availability of multi-superior programming languages today, designers can easily design apps with such features. This paper focuses on the software application that is used to encourage verbal communication in the intervention for children with ASD. The aim is to determine the minimum requirements that a verbal communication intervention app should have to satisfy children with ASD while also being helpful to caregivers and therapists in terms of monitoring purposes. 

## 2. Methodology

Only one research question was formulated in this review: how should a verbal communication intervention app be designed to accommodate children with ASD, caregivers, and therapists? Relevant peer-reviewed journal articles were collected and analyzed to obtain information on the apps and intervention procedures. Detailed analysis on both subjects will reveal the limitations and strengths of each study, which will be extracted to answer the research question. 

This review was conducted by following the PRISMA guidelines [23]. Searches were carried out through electronic sources in the following databases: Scopus, Springer, PubMed, Education Resources Information Centre, and Google Scholar. Search terms used include combinations of all or some of the following free-text terms and Boolean operators: autism, children, intervention, software, app, technology, and verbal communication. Only English-language articles were selected due to the lack of resources for translation, and the search was limited to articles published within the last ten years (2012 to 2021). 

**Figure 1:**
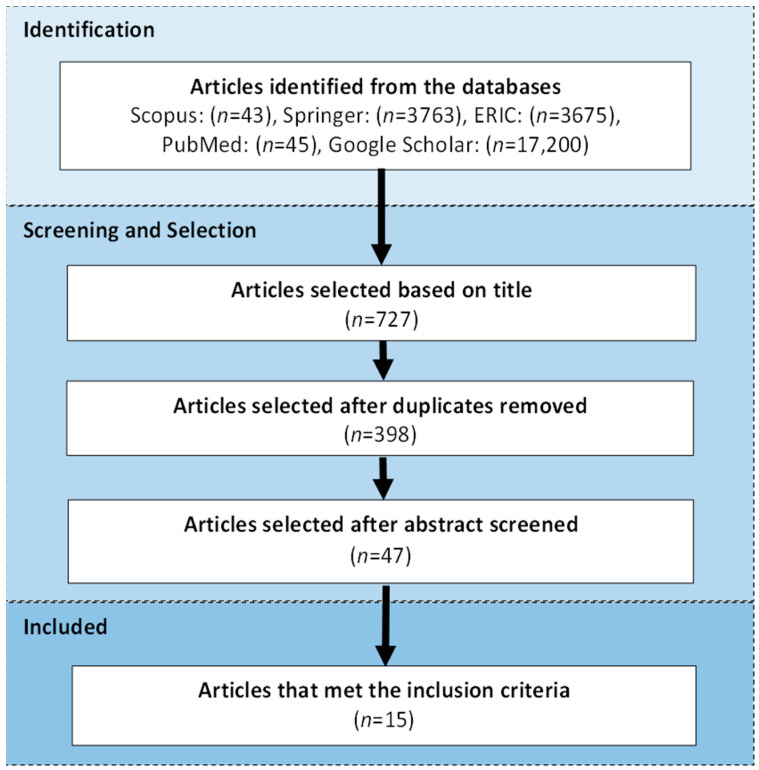
The searching and selection process.

The search and selection process is described in Figure 1. The searching stage was conducted in two sets. The first set was performed using all keywords with the “AND” or “OR” operators. The second set used the keywords: autism, children, intervention, and verbal communication with one of the following keywords: software, app, and technology at a time. For the databases that produced more than 1000 results, only the articles in the first ten pages were considered. The initial selection was made based on the title (*n* = 727). Duplicates were removed (*n* = 329), and the abstracts of the remaining articles were examined. The abstracts that did not mention software or app, autism, children, and intervention were excluded (*n* = 351). The remaining articles underwent a full-text review (*n* = 47). Finally, the articles that met the following inclusion criteria were selected (*n* = 15):

- At least one child (16 years old and below) with ASD was included in the study;
- The application software that was used as the intervention tool during the experiment;
- The focus of intervention was verbal communication or verbal interaction.

## 3. Results

A total of fifteen articles that met the inclusion criteria are listed in Table A1 (Appendix A). The following information was extracted from these fifteen articles: participants’ information, devices and application software, the focus of intervention, intervention procedures, and intervention outcomes. 

### 3.1. Participants’ Information

Altogether, 179 participants between the ages of two and sixteen years were involved in the selected studies. Of them, 12 participants were typically developing (TD) children in which they were used as the benchmark. A total of 127 participants were diagnosed with ASD, and 40 were identified as having cognitively low functioning or other developmental disabilities (i.e., multiple disabilities, intellectual disabilities). Table 1 summarises the information of the participants involved in those studies.

### 3.2. Devices and Application Software

The iPad was the most used device, followed by a tablet, phone, and computer as the teaching tools with 80.0%, 13.3%, and 6.7%, respectively. In terms of application software, three studies used custom-made apps: iCAN, Turkish sequencing game, FindMe (Autism), and SIGUEME, while the rest used readymade apps. The most used readymade app was the Proloquo2Go app, where three studies used it to teach the participants to make valid requests for the preferred object. Table 2 lists the devices and apps used in the fifteen studies.

### 3.3. Focus of Intervention

The fifteen selected studies focused on improving verbal communication and interaction as the intervention’s key target, summarized by Table 3. A study focused on teaching imitation skills to improve receptive and expressive language skills among children with ASD. A total of five studies focused on teaching requesting skills using verbal phrases: “I want __.”, “I would like __.”, or “__ please” phrases, sign languages, and technology-based aid. For participants with limited verbal speech, the intervention was conducted using sign language and sign-like gestures. For example, they used the sign-like gesture “no” by shaking their head and saying “no-no” to indicate that they did not want to do something. A technology aid like the specific “*I want*.” icon in the iPad was used to teach individuals with limited or no speech to make the correct request. 

Of the examined studies, two focused on social interaction among children with ASD and their peers or caregivers. These studies aimed to motivate children with ASD to make social comments or expressions and improve their verbal abilities. A total of three studies focused on vocal expressions during social interactions to describe the objects, such as “it is a worm.”, to explain the process out loud during working. In addition, exclamations (e.g., “wow” and “oh no”) and any social comments like “I like it.”, “I am sorry.” and “Thank you.” were also used to communicate. 

Another study focused on sequencing story event skills, an essential component in expressive language skills that is frequently absent in children with ASD. The skill of sequencing is one of the basic skills underlying communication, reading, and speaking. The lack of sequencing story events skills limits the use of language that promotes interactions, which results in a further decrease in opportunities to engage in a meaningful communication experience. The remaining studies were aimed at general social communication to increase the frequency of verbal communication. 

### 3.4. Intervention Procedure

Cardon conducted a study to determine the functional relationship between caregiver implemented Video Modeling Imitation Training (VMIT) with increased imitation skills in young children with ASD [24]. The VMIT is a new protocol that is designed to teach young children with ASD to imitate skills using the iPad. Conceptually, VMIT supplements video models that include specific prompt and praise procedures that are similar to those used in a clinical setting. To begin, the caregiver shows the pre-recorded clips (video) of the one-step actions to the child. Then, the child is given 10-s to imitate the action they had just viewed. The caregiver will praise the child if they imitate the action and show the following clip. Otherwise, the same clip is shown again and then paused. If the child fails to imitate the action after three trials, the caregiver will physically prompt the child to perform the action and provide verbal praise before moving on to the following clip. The location of the experiment took place in the University autism laboratory for the pre-assessment session and participants’ homes for baseline, treatment, post-assessment, and follow-up sessions. 

Chien et al. conducted a study to investigate the effectiveness of the iCAN application [25]. It is a tablet-based system that adopts the successful aspects of the traditional PECS approach to improve the communication skills of children with ASD. The iCAN was designed as a teaching-assistive tablet application for parents and teachers to teach functional communication skills. The iCAN consists of three interactive modes: (1) picture card and sentence interaction, (2) card creator and editor, and (3) commonly used sentences practice. The caregivers were asked to use the iCAN system to teach their children two to five times weekly for four months in the study. Caregivers were provided with a diary to collect metadata, such as notes, comments, and descriptions of their system usage.

Desai et al. conducted a study to investigate the iPad as an alternative communication device in delivering intervention for students diagnosed with ASD [26]. The 16-week sessions were designed based on the technology delivery protocol [27], which included an initial assessment, access technology introduction, teaching (staff and family), and training (student). Doenyas et al. conducted a pilot study to investigate the reaction and interest of children with ASD to educational iPad apps [28]. In the study, the researchers developed a web-based game that aimed to teach sequencing story events skills. They implemented the prompt and prompt-fading procedures of Applied Behavioral Analysis (ABA) in their app. The app has “testing” sessions with no prompts or rewards and “teaching” sessions with prompts, rewards, and demonstration of correct responses. The procedures of their experiment were baseline–testing, intervention–teaching, and post-intervention–testing. 

Dundon and McLaughlin conducted a study to evaluate the effectiveness of employing the Model, Lead and Test (MLT) error correction procedure across iPad applications during the intervention to teach proper communication [29]. In the model stage, a teacher or trainer modeling the correct response. During the lead stage, the student and teacher correctly respond together while the student must independently complete the task correctly in the test stage. The MLT design was employed in the study with the selected iPad applications (i.e., My Choice Board and GoTalk NOW Free) to teach correct requesting skills. Multiple baseline design was used to evaluate the effectiveness of the iPad for communication. The dependent variable was the number of valid requests the participant made using the iPad. The procedures were as follows: (i) baseline, (ii) My Choice Board + MLT, (iii) My Choice Board: independent, (iv) GoTalk NOW Free + MLT, and (v) GoTalk NOW Free: independent. 

Fletcher-Watson et al. reported developing the FindMe (Autism) application for children with ASD to rehearse key social communication in a motivating and rewarding environment [30]. The app consists of Part One: Attending to Other People and Part Two: Following Social Cues. Three experiments were conducted to investigate the functionality of their app: (1) User testing with TD toddlers, (2) Single session user testing with toddlers with ASD, and (3) User testing with children with ASD and their parents at home. 

Flores et al. investigated the iPad as a communication device by comparing it to the traditional communication system using picture cards during the intervention [31]. The study was conducted for three hours each day across five days per week over five weeks. The data that were collected in the study were the frequency of communication under two conditions: (1) picture-based condition, using picture communication symbols, and (2) iPad condition, Pick a Word with iPad. The procedures were implemented in the following order: 1-2-1-2-1. 

In their intervention procedure, a study by King et al. applied the adapted picture exchange communication system (PECS) protocol with iPad and Prolonquo2Go [32]. Initially, the iPad1 with the Proloquo2Go application was designed to function as a SGD. The study aimed to determine whether a child with ASD can acquire the necessary skills to request preferred items on the iPad using the Proloquo2Go application. The PECS framework was adapted and modified to be used with the iPad and Proloquo2Go application during the intervention phases. In the adapted PECS, only four phases of the PECS protocol were included, and the procedure of each phase was modified to accommodate the Proloquo2Go and iPad. The experiment included datasheets for (1) a six-stimuli paired choice preference assessment, (2) individual phases, (3) procedural fidelity checklists, and (4) the reinforcer assessment for individuals with severe disabilities (RAISD). The RAISD was administered in an interview format to the lead teacher or the parents for the preference assessment to identify the preferred items. A total of six items were chosen to be included in a six-stimuli paired stimulus (forced-choice) preference assessment. The data were measured based on the independent responses that were made by the participants in six phases: baseline, P1, P2, P3a, P3b, and P4. This study provides preliminary empirical evidence for implementing technology with the modified and adapted pre-existing teaching mechanisms (e.g., PECS protocol) to establish a requesting repertoire for children with ASD. Table 4 defines the independent response used in each phase.

Plavnick et al. conducted a study to evaluate the combination of contingent reinforcement and match-to-sample (MTS) instruction with a reading application, entitled Headsprout Early Reading (HER), in the intervention [33]. The MTS instruction was developed to teach negation. It consisted of four levels and utilized graduated guidance to prompt participants to match stimuli to the vocal instructions that were associated with negation. The correct interactions per minute and progression through the intervention program were calculated. 

In 2014, Roche et al. implemented the initial SGD intervention (Phase 1) [34]. Then, an alternative SGD, which is a software application with technology devices (Proloquo2Go with iPad), was used to teach requesting skill talk to children with neurodevelopmental disorders like ASD. In the experiment based on [35], all participants had attended a 60-min clinical session once each week for two months. During each session, participants underwent a sequence of phases, which included baseline, intervention, maintenance, and generalization. The procedures for each phase were based on the specific instructional procedures that were designed in [36], which included behavior chain interruption, graduated guidance “prompting techniques.”, time delay, and differential/natural reinforcement. 

Vélez-Coto et al. investigated the effectiveness of technology-based intervention for children with low-functioning ASD to perform academic tasks and improve their abilities and knowledge [37]. The research group has developed an application named SIGUEME. This app has six phases with different exercises: (1) attention, (2) video, (3) image, (4) drawing, (5) pictogram, and (6) games. Each phase was conducted in three or four sessions using videos or images to stimulate visualization, abstraction, generalization, and association of children with ASD. The exercises were performed using three modes: watching mode (the exercises are performed automatically), acting mode (the exercises require user interaction to be performed), and guessing mode (require the correct user interaction to be performed). Table 5 summarizes each phase in the intervention.

The study employed a “pre-test/post-test” design with two non-equivalent groups to test the effectiveness of using this app in intervention. The experimental group underwent intervention using the SIGUEME. The control group used the everyday school materials: PECS, PowerPoint presentations prepared by the tutors, and videos with images of natural objects displayed on tactile devices or interactive whiteboards. The performance was measured based on the students’ behaviors during the intervention by using the assessment questionnaire that was composed of five factors: attention, recognition, association and categorization, interaction, and communication. The intervention sessions are shown in Table 5.

In 2012, a study was conducted to investigate the actions (especially attention, communication, interaction, and creativity) of children with ASD in a strength-based learning environment with multiple technologies [38]. This study was a part of the CASCATE research project. In this study, the researchers focused on three technology-based workstations: (1) building with bricks, (2) symbol matching, and (3) storytelling. The activities that were provided in the workstations included tasks on a computer, as shown in Table 6.

Waddington et al. conducted a study that used an iPad as a speech generating device (SGD) in the intervention [39]. Their intervention aimed to teach the children with ASD to engage in multi-step communication sequences using the iPad. The multi-step sequence involved: (1) making a general request for access to a toy/drawing materials, (2) making a specific request for one of two preferred toys/sets of materials, and then selecting the corresponding item, and (3) saying thank you after accessing the requested item. The experiment phases involved: (i) static baseline, (ii) static intervention, (iii) generalization, and (iv) follow-up. The procedures for each experimental phase were similar, which included the three-step sequence with a time delay of 10-sec for each sequence. Systematic instructional procedures were used for the static intervention phase to implement the three-step sequence [36].

In 2014, a study was conducted to examine the effects of using the iPad to assist students with ASD in learning expressive communication skills [40]. A total of three types of communication were included in the study: (1) request, using “I want __” phrase to request a preferred item, (2) respond, answering questions from their caregivers, and (3) social comments, like “That was fun” after completing a task. Data were collected during two 10-min sessions, 2-days per week for 2 to 3 weeks for the baseline assessment. For intervention, data collection was continued in the same sessions 2-days per week for 6-weeks. Instruction was provided using the least-to-most prompting hierarchy after a 5-sec pause.

The summary of the procedures that were used in the fifteen selected studies is shown in Table 7. Of these, nine studies employed evidenced-based procedures in their intervention, while the rest did not describe the details.

### 3.5. Intervention Outcomes

Findings from the selected studies indicated that the app-based intervention through electronic devices (i.e., iPad, tablet, computer) could help children with ASD to learn social communication skills and improve their social interaction with their peers and caregivers. The app-based intervention motivates the children to make a social response, such as saying “thank you”, naming objects, answering questions, and reading text aloud. The children also learned requesting-skill talk phrases, like “I want” or “please” to ask for a preferred item, and they improved their skills in motor imitation, expressive and receptive language, and sequencing.

Using the application software resulted in faster learning than picture-communication symbols [33]. A total of four studies showed increased verbal interaction upon introducing application software as the intervention tool and could motivate children with ASD to communicate verbally. Roche et al. suggested that the iPad-based SGD motivates children with ASD to communicate in the early intervention phase [34]. Their study implemented the “initial SGD intervention, followed by removing the SGD” approach to promote functional communication among the children with ASD. There was an increase in natural speech production during intervention for both phases: Phase 1—with the iPad and Phase 2—removing the iPad. The researchers suggested that the initial use of an alternative model, such as an iPad-based SGD, could increase the level of motivation to communicate and ensure a high rate of success to facilitate learning as valid responses could easily be prompted. 

Studies also suggested that communication-assistive apps could increase the frequency of natural speech production in some children with ASD. The increased frequency of natural speech production during the intervention was an interesting result considering the children’s many communication difficulties. King et al. suggested that apps could be used with children with limited vocal output to acquire a requesting repertoire [32], however, it may also assist in increasing verbal requests for children with ASD. Additionally, children were motivated to interact with the application by reading aloud text, naming or describing objects, and making sounds of the things that were displayed in the application.

In addition, the implementation of application software could promote independent learning among individuals with ASD. A total of six studies showed that participants with ASD could perform the given tasks in the electronic devices independently without constant one–one adult support. With their high interest in the devices, they were motivated to learn and complete the given tasks in the devices. Desai et al. stated that external prompting steadily decreased after introducing technology devices [26]. However, the increased use of external guidance and reinforcers through teacher-led instruction would be necessary for individuals with poor fine motor skills before completing the task independently [28]. Likewise, the increased use of external support (e.g., ongoing one–one adult support), especially for the young children, would be needed to guide them on using the devices before intervention. Implementing a concentrated procedure is necessary for children who could not learn how to use the apps independently. Caregivers could also motivate the children to engage with the apps by showing examples [33]. Table 8 lists the intervention outcomes.

## 4. Discussion

The apps used in the fifteen selected studies were not explicitly developed for the verbal communication intervention. The Proloquo2Go, My Choice Board, GoTalk NOW, Sonoflex, Pick A Word, and iCan apps were designed to function as the AAC or SGD. These apps on their own were unable to encourage the user to initiate verbal communication. Instead, the apps talk on their behalf. On the other hand, the Turkish sequencing game, SIGUEME, Find Me (Autism), HER, iMovie-VMIT, Symbol matching, and Picture-based computer apps were developed as cognitive or social educational tools. Similarly, these apps will not encourage verbal communication if used in their original design. 

The readymade apps (Proloquo2Go, My Choice Board, GoTalk NOW, Sonoflex, Pick A Word, HER, iMovie-VMIT, Symbol matching, and Picture-based computer) contain adequate tools or exercises, thus work reasonably well for their specific purposes only. On the contrary, the custom-made apps (iCAN, Turkish sequencing game, SIGUEME, and Find Me (Autism)) contain inadequate tools or lessons. Due to these limitations, the readymade and custom-made apps used in the studies could not effectively perform as verbal communication intervention tools. The researchers had to apply additional procedures when using the apps to deliver the interventions in their studies. The approaches that were taken by the researchers were analyzed to deduce the features of a satisfying verbal communication intervention app. Detailed analysis reveals that the intervention apps should be designed with the following characteristics:

- Attractive and engaging to the children with ASD;
- Able to identify the child’s capability and suggest appropriate lesson activities;
- Encompass specific learning outcomes with multilevel lesson strategies;
- Use systematic evidence-based intervention procedures in the activities;
- Are able to evaluate the child’s learning progress;
- Allow the caregivers or therapists to keep track of the application usage and performance.

Most of the participants in the selected studies showed high levels of interest in the apps during the intervention. They showed a preference for electronic devices compared to traditional materials like books and cards. The high level of interest in electronic devices could motivate children with ASD to stay engaged in learning. No challenging behaviors were observed upon the introduction of the apps during the intervention. However, the features and contents of the apps, particularly how interesting the tasks were to the children and the novelty of the tasks, influenced children’s motivation to communicate verbally [41]. If the software application did not respond as expected, the children would react by making sounds to express their annoyance. 

Children with ASD process icons and images faster than texts [42], and they respond to exciting pictures that are displayed on the apps. An additional feature like voice output can help to increase children’s attention and convey the message. The voice output could motivate the children to ‘voice over’ (or speak out loud), especially those individuals with little control over their vocalizations [43]. Imitating the voice from the device is the first big step for the children to initiate speech and learn verbal communication skills. Unfortunately, finding an app that ‘speaks’ their mother-tongue language is often challenging [44]. In addition, the apps could be further enhanced by integrating the speech processing techniques for detecting correct verbal responses to encourage verbal communication [45].

A good intervention app should be equipped with a preliminary assessment section to identify the child’s capability and suggest practical lesson activities accordingly [30,46]. It would be helpful if the apps could indicate the specific competencies that the child should have to begin. A multilevel lesson strategy for each learning outcome should be provided as individuals with ASD have different learning abilities. Some intelligent procedural modifications should be in place, in which the implemented instructional procedures can be individualized to suit the learning characteristics of every child with ASD. 

Interventionists’ systematic evidence-based intervention procedures should be adapted into the software application as they were proven effective [47]. For example, the adaptation of the ABA concept by Doenyas et al. was proven to improve the children’s expressive language and social communication behaviors [28]. The researchers also suggested that implementing MLT procedures could help promote independence and correct requesting during the intervention [26,29]. Parsons suggested that the 3T (theory, technology, and thoughts) design approach should be taken as a framework for designing and developing future ASD-specific technology apps [48]. Fletcher-Watson et al. stated that it is essential to include evidence-based intervention procedures in the apps that are designed for individuals with ASD [30]. 

According to the professionals within the ASD community, the intervention apps should have a system to evaluate the child’s learning progress and a reporting system for the caregivers and therapists [49,50]. It will be an added value if the application software is interactive. For instance, it sends progress notifications, allows users to add their voices, is intelligent enough to allow sufficient time to carry out the learning activities, and monitors and assesses the proposed activities. The apps, aided with intelligent algorithms, could adjust the learning strategy depending on the child’s performance.

The above findings were deduced by analyzing each intervention approach in the fifteen selected studies. It can be observed that the researchers had taken additional measures in the intervention process due to the limitations of the apps. In all studies, the researchers concluded that the apps were contributing positively to the target participants. However, most studies used a small sample size, consisting of one to ten children with ASD. Only three studies had more than ten participants. All studies did not systematically analyze the intervention duration, but they emphasized that the frequency and duration of intervention play a significant contribution. In accordance, this paper has excluded the intervention frequency and duration as factors to be stressed when examining the studies. We believe a systematic and comprehensive analysis should be conducted using proper intervention apps to get the optimum intervention frequency and duration.

## 5. Conclusions

This technology could enhance the intervention quality as it enables correct responses to be easily prompted without fatigue. Technology that is paired with human interventionists could contribute to better reinforcement which facilitates learning and motivates verbal communication even in low-functioning children with ASD. With a proper schedule, the use of technology can make the parent-delivered interventions at home more focused and systematic. The continuous use of interventions in a home environment contributes to longer treatment times that would improve the child’s ability to communicate. Additionally, the application software with technology devices (i.e., iPad, tablet, computer) could be cheaper than augmentative and alternative communication (AAC) devices and they could be useful and powerful instruments for intervention. Finally, the use of technology in intervention does provide benefits to children with ASD. However, it should never replace the interventions that are provided by qualified therapists.

## Figures and Tables

**Table 1:** Participants’ information.

Diagnosis																
Age(Years)	2	3	4	5	6	7	8	9	10	11	12	13	14	15	16	=
TD			12 ^6^													12
ASD	2 ^1^	1 ^1^	3 ^1,4,11^	1 ^11^	3 ^9^	1 ^14^	3 ^7,14^	1 ^7,10^	1 ^14^	1 ^4^				1 ^4^		50
			20 ^6^						11 ^2^							
ASD with Cerebral palsy												1 ^3^				1
ASD with limited communication skills		1 ^8^	2 ^5,8^	1 ^8^			1 ^13^	1 ^13^	3 ^15^	1 ^13^	1 ^13^					11
ASD or ASD with cognitively low functioning		65 ^12^														65
Cognitively low functioning		37 ^12^														37
Other developmental disabilities		1 ^10^														3
								1 ^7^		1 ^7^						
Total = 179 (127 children with ASD)																

The superscripted numbers refer to the articles listed in Table A1. TD = Typically Developing. ASD = Autism Spectrum Disorder.

**Table 2:** Devices and application software used for intervention.

Application Software	Description	*n*
iPad		
Proloquo2Go	A speech-generating device (SGD). Press preference item on the iPad with the “I want.” icon to make a request.	3 ^8,10,11^
Turkish sequencing game	Sequencing card game application in the Turkish language. Five different sequencing stories: brushing teeth, making a sandwich, getting on and riding a bike, taking a jacket out of the closet and putting it on, and pouring orange juice into a cup and drinking it.	1 ^4^
My Choice Board	Audio-visual display of options so users can express specific needs and wants by touching the icon.	1 ^5^
GoTalk NOW	Customisable augmentative and alternative communication (AAC) app for iPad.	2 ^3,5^
FindMe (Autism)	Designed to help young children with autism to practice simple social skills. It consists of two parts: attending to other people and following social cues.	1 ^6^
Sonoflex	AAC vocabulary app that turns symbols into clear speech.	1 ^15^
iMovie-VMIT	Video editing software application adopting the Video Modelling Imitation Training (VMIT).	1 ^1^
Pick A Word	Required user to touch a color photograph on the screen to make a request. Each item or request is depicted on the screen in a photograph. The I-WANT picture was a photograph of open hands depicting the American Sign Language sign for ‘I want.’	1 ^7^
SIGUEME	Consist of six phases with different exercises each one, which range from Attention, Video, Image, Drawing, Pictograms, and Games.	1 ^12^
Tablet		
iCAN	A tablet-based system that adopts the successful aspects of the traditional PECS.	1 ^2^
HER	A behavior analytic online reading program.	1 ^9^
Computer		
Symbol matching *	User is required to match sounds to visual symbols, pictures of emotions, shapes, the number of objects in a photo to numerals, and recognizing hidden objects.	1 ^13^
Picture-based computer application *	User is required to create stories by dragging and dropping pictures in the story’s timeline.	1 ^13^

* Application not explicitly stated in the paper. The superscripted numbers refer to the articles listed in Table A1. PECS = Picture Exchange Communication System. HER = Headsprout Early Reading.

**Table 3:** Focus of intervention.

Target Behavior	Number of Articles
Imitation skills	1 ^1^
Requesting skills	
Select the specific icon	3 ^5,8,11^
Verbally	2 ^10,15^
Sequencing skills	1 ^4^
Sign language	1 ^13^
Social Response	
Respond to questions	1 ^15^
Oral reading	1 ^9^
Social interaction	
Vocal expression	1 ^13^
Social comments	2 ^14,15^
Social communication	6 ^2,3,6,7,12,13^

The superscripted numbers refer to the articles listed in Table A1.

**Table 4:** Independent response for each phase.

Phase	Independent Response
Baseline, P4	Independently pressing the icon on the iPad screen consistent with the item indicated as a preference plus the “I want.” icon, then the message window
P1, P3a, P3b	Independently pressing the icon on the iPad corresponding to the preferred tangible or edible item indicated as a preference
P2	Independently pressing the icon on the iPad corresponding to the preferred tangible or edible item indicated as a preference after traveling

**Table 5:** Intervention sessions in [36]. S = Number of sessions, V-video, I-image.

Phase	S	Tool	Mode
Attention	4	V	“watching”
Attention	4	V	“acting”
Video	3	V	“acting”
Image	3	I	“guessing”
Drawing	3	I	“guessing”
Pictogram	3	I	“guessing”
Games	4	I	“guessing”

**Table 6:** The activities in the workstations [38].

Workstation	Activities
Building with bricks	Lego construction from the model on the computer application
Symbol matching	Match symbol from the computer application by pressing the tile with a hand (tiles on the table or floor) or foot (tiles on the floor)
Storytelling	Create a story by using a picture-based computer application and a touch screen.

**Table 7:** Summary of the intervention procedures.

Procedure	No. of Studies
ABA-based	1 ^4^
PECS—modified to adapt to technology	1 ^8^
Video modeling instruction	1 ^1^
Match to sample instruction	1 ^9^
Least-to-most prompting sequence	2 ^14,15^
The instructional procedure by Duker et al. (2004)	2 ^11,14^
Technology delivery protocol by Mumlord et al. (2014)	1 ^3^
A procedure designed by author/s:	
Model, Lead, Test (MLT) error correction procedure	1 ^5^
No specific instruction	6 ^2,6,7,10,12,13^

The superscripted numbers refer to the articles listed in Table A1.

**Table 8:** Intervention outcomes.

Findings	*n*
Improved communication skills	2 ^7,14^
Improved interaction	2 ^12,15^
Increase correct interaction (read text out loud)	1 ^9^
Increase independency	6 ^1,3,4,5,6,9^
Increase motivation in learning	3 ^2,6,12^
Increased natural speech production	3 ^2,10,15^
Increased number of vocal expressions or signs (or sign-like gestures)	2 ^2,13^
Increased sequencing skills	1 ^4^
Increased verbal requests	2 ^8,15^
Increased level of imitation	1 ^1^
Make correct requesting	6 ^5,8,10,11,14,15^
Positive gains in expressive and receptive language skills	1 ^1^
Reduce negative behaviors like reaching or hitting	1 ^11^

The superscripted numbers refer to the articles listed in Table A1.

## Appendix A

**Table A1:** Summary of research articles; n(x; y) = number of participants (Age in years; Diagnosis).

(Authors, Year)	Participants	Device; Application	Target Behavior	Intervention Procedure	Intervention Results
^1^ (Cardon, 2012) [24]	*n* = 4 (1–4; ASD)	iPad; iMovie-VMIT	Imitation skills	(i) baseline sessions, actions were presented live by the caregiver, and (ii) VMIT implementation, actions were presented from recorded clip made by the caregiver. Procedures: action was presented—paused for 10 s and wait for the child to imitate the action—(i) if imitated, the child received verbal praise from the caregiver; (ii) else, the action was shown again. After the third trial, the caregiver physically prompted the child to act. Then, the subsequent action was presented.	All four participants made positive gains in expressive communication and motor imitation Three out of four participants demonstrated gains in auditory comprehension
^2^ (Chien et al., 2014) [25]	*n* = 11 (5–16; ASD)	Tablet; iCAN	Communication skills	The iCAN consists of three interactive modes: (1) picture card and sentence interaction, (2) card creator and editor, and (3) commonly used sentences practice. Caregivers were asked to use the iCAN system to teach their respective children two to five times weekly for four months. Caregivers were provided with a diary to record notes, comments, and descriptions of their usage of the system.	Participants were more expressive and able to communicate their needs more clearly And significantly improved their verbal abilities
^3^ (Desai, Chow, Mumford, Hotze, & Chau, 2014) [26]	*n* = 1 (13; ASD and Cerebral palsy)	iPad, GoTalk NOW	Communication skills and non-academic functioning	Involved 16 weeks protocol including initial assessment, access technology introduction, and training of the student, teaching staff, and family with the iPad. Outcome measures were administered selectively at each assessment point. The measures include (i) Quebec User Evaluation of Satisfaction with assistive Technology (QUEST), (ii) Goal Attainment Scaling (GAS), (iii) communication Matrix (CM) assessment tool, and (iv) School Function Assessment (SFA) [27].	Decrease in the number of prompts required (increase independence) Participants demonstrated gains in all areas of early language development, from 10% to >30% of percentage score after the intervention.
^4^ (Doenyas, Şimdi, Özcan, Çataltepe, & Birkan, 2014) [28]	*n* = 3 (4, 11, 15; Autism)	iPad; Turkish sequencing game	Sequencing story event skills	There were two sessions included in the sequencing game app: (i) testing session, to measure the child’s independent performance using the iPad, and (ii) teaching session, which included prompts and reinforcers. ABA design was used in the study. Procedures: (i) baseline, the testing session was used with no prompt, (ii) intervention, teaching session, and (iii) post-intervention, same procedure with baseline.	All participants showed improvement in sequencing skills with the highest average scores during intervention and post-intervention compared to the baseline.
^5^ (Dundon & McLaughlin, 2013) [29]	*n* = 1 (5; ASD with limited communication skills)	iPad; My Choice Board, GoTalk NOW Free	Requesting skills	The intervention follows the Model, Lead and Test (MLT) error correction procedure, (i) M: teacher or trainer modeling the correct response, (ii) L: student and teacher correctly respond together, and (iii) T: student independently complete the task correctly. Multiple baseline design. Procedures: (i) baseline, (ii) My Choice Board + MLT, (iii) My Choice Board: independent, (iv) GoTalk NOW Free + MLT, (v) GoTalk NOW Free: independent.	Findings indicated that the number of valid requests increases during the apps-based intervention phases compared to baseline.
^6^ (Fletcher-Watson, Pain, Hammond, Humphry, & Mcconachie, 2016) [30]	*n* = 12 (6; TD) *n* = 10 (4–5; ASD) ^M2^ *n* = 10 (4–6; ASD) ^M3^	iPads; FindMe (Autism)	Simple social skills	The FindMe (Autism) application has two parts, (i) attending to other people—teaching the children with ASD to prioritize other people in a scene, (ii) following social cues—similar to the first part, except the character was re-located to a series of shop interiors. Three experiments were conducted, M1: User testing with TD toddlers, M2: Single session user testing with toddlers with ASD, and M3: User testing with children with ASD and their parents at home.	Results showed high levels of interest in the app among a sample of children with autism M2: Verbal responses were common M3: Parents reported a positive impact of the iPad
^7^ (Flores et al., 2012) [31]	*n* = 5 (8, 9, 11; ASD and other developmental disabilities	iPad; Pick a Word	Communication behaviors	The experiment was to identify the frequency of communication under two conditions: (A) picture-based condition: picture communication symbols, and (B) iPad condition: Pick a Word with iPad. The procedures were implemented in the following order: A-B-A-B-A	Results show an increase in communication behaviors with the iPad. However, there was no clear pattern across all participants
^8^ (King et al., 2014) [32]	*n =* 3 (3, 4, 5; ASD with limited to no vocal output)	iPad; Proloquo2Go	Requesting skills by using the phrase “I want ___” (Phase 4 of the PECS protocol)	They adapted and modified the PECS framework for the iPad and Proloquo2Go application during the intervention phases. The experiment procedures include (i) baseline probes, (ii) iPad + Proloquo2Go application training, (iii) prompting, and (iv) adapted PECS: Phase 1, Phase 2, Phase 3a, Phase 3b, and Phase 4.	All participants managed to achieve mastery criteria of the adapted PECS from Phase 1 to Phase 3a. Vocal requests were observed to have increased for all participants
^9^ (Plavnick, Thompson, Englert, Mariage, & Johnson, 2016) [33]	*n =* 3 (6; ASD)	Microsoft Surface RT tablet; HER	Oral reading skills	Correct interactions per minute (CIPM) were used as the primary dependent variable. CIPM calculates the number of accurate responses with the HER application. Procedures include baseline and intervention with (i) contingent reinforcement and (ii) match-to-sample instruction.	Participants demonstrated an increase in CIMP during the intervention. Two participants could independently complete HER episodes in less than 40 intervention sessions
^10^ (Roche et al., 2014) [34]	*n* = 2 (9, 3; ASD and general development delay)	iPad; Proloquo2Go	Requesting skills	Single-case experimental designs Participants received three sequential phases: (a) baseline, (b) SGD intervention (the procedures were identical to the baseline except that requesting method was changed to SGD-based request), (c) removal of SGD (similar procedures as in the previous phase but SGD was removed).	Increase the use of natural speech to request the preferred item during the SGD intervention phase and at the final phase.
^11^ (Sigafoos et al., 2013) [35]	*n* = 2 (4, 5; autism)	iPad; Proloquo2Go	Requesting skills	The sequence of phases for the intervention: baseline, intervention, maintenance, and generalization. For each phase, the specific instructional procedures were implemented, which include: (a) behavior chain interruption, (b) graduated guidance “prompting techniques.” (c) time delay, and (d) differential/natural reinforcement	Findings showed that correct requesting occurred during the intervention (i.e., consistent and steady requesting and other behaviors like reaching and hitting did not occur)
^12^ (Vélez-Coto et al., 2017) [37]	*n* = 78, 28 (3–16; ASD and, or cognitively low functioning, Level 1 and 2)	iPad or tablet; SIGUEME	Attention, recognition, association and categorization, Interaction, and communication	Pre-test and post-test design with non-equivalent groups: (i) experiment group—intervention with SIGUEME, and (ii) control group—intervention with everyday school materials: PECS, PowerPoint presentations, and videos with images of natural objects Data collection was conducted using a questionnaire comprises of 22 items completed by their caregivers. Confirmatory factor analysis (CFA) was conducted to test the fit of the expert-compiled assessment questionnaire.	Improve in attention, association or categorization, and interaction in low functioning children with ASD.
^13^ (Vellonen, Kärnä, & Virnes, 2012) [38]	*n =* 4 (8, 9, 11, 12 old; Autism with limited verbal language skills)	Computer; (i) Duplo or LEGO bricks, (ii) Symbol matching, and (iii) Picture-based computer application	Communication in the form of speech	A technology-enhanced environment was set up, which included three technology-based workstations: (i) building with bricks, (ii) symbol matching, (iii) storytelling The primary data were collected by videotaping the children working with their teachers or school assistants at the workstations. The additional data were collected by observing the sessions.	Increase in communication and interaction during activities in the workstation with the number of children’s vocal expressions, N_vocal_ = 1020 and number of signs and sign-like gestures N_sign_ = 37
^14^ (Waddington et al., 2014) [39]	*n* = 3 (7, 8, 10; ASD)	iPad; Proloquo2Go	Requesting using multi-step communication	The experiment phases involved: (i) static baseline, (ii) static intervention, (iii) generalization, and (iv) follow-up. One participant went for the progressive baseline and intervention as little progress was observed during baseline. Measurement for correct three steps in communication sequences was as follows: S1-general request within 10 s, S2-specific request for one of two preferred toys within 10 s, and S3-communicate a social response (i.e., “Thank you.”) upon receiving requested item.	Participants managed to complete the three-step sequence during the static intervention phase and the following phases
^15^ (Xin & Leonard, 2014) [40]	*n* = 3 (10; ASD with limited speech and language abilities)	iPad; Sonoflex	Expressive communication	A single subject, multiple-baseline design with AB phases, (A) Baseline, and (B) Intervention The total number of occurrences in each type of communication, (i) responses, (ii) requests, and (iii) social comments during AB phases, were calculated.	All participants showed improvement in their communication skills (increase in the initial request, responses to questions, and making social comments)
